# Comparative Analysis of 16 Aging Concepts and Their Influence on Aging Narratives: Bibliometric and Content Analysis

**DOI:** 10.2196/72011

**Published:** 2025-09-23

**Authors:** Na Xiao, Bo Xia, Laurie Buys, Connie Susilawati, Martin Larbi, Qing Chen

**Affiliations:** 1 School of Architecture and Built Environment, Faculty of Engineering, Queensland University of Technology, D Block Level 4 401, Gardens Point, Brisbane, 4000, Australia, 610481759056; 2 Faculty of Health Sciences, Australian Catholic University, Brisbane, Australia; 3 School of Economics and Finance, Faculty of Business and Law, Queensland University of Technology, Brisbane, Australia

**Keywords:** aging concepts, health aging, successful aging, active aging, bibliometric analysis

## Abstract

**Background:**

Globally, various aging concepts (such as healthy aging, successful aging, and active aging) have emerged to promote the goal of “aging well” and have gained widespread attention in academia, policy, and practice to change the negative narrative on aging. However, whether and how these aging concepts have contributed to changing the negative narratives remains unclear. Moreover, they are not clearly defined nor widely agreed upon, often creating ambiguity and confusion.

**Objective:**

This paper aims to provide a comprehensive review and comparative analysis of 16 aging concepts, with a particular focus on how their evolution in research has contributed to shifting the narrative surrounding aging.

**Methods:**

This study used the bibliometric software VosViewer (Center for Science and Technology Studies) to visualize international collaboration among countries and cocitation networks among journals. This helped identify which countries and journals play central roles in research on aging concepts and revealed how academic contributions are distributed globally. Additionally, content analysis supported by the corpus linguistics software AntConc (Waseda University) was conducted to examine and compare the main focuses, applications, challenges, and future research directions of these concepts.

**Results:**

The findings indicate that while all 16 aging concepts share the common goal of improving the quality of life for older adults, they offer different perspectives, encompassing health management, social participation, mental health, and technological innovation. Key challenges to achieving the goal of each aging concept were identified, including unequal access to health care resources, barriers to social participation, and difficulties in adopting technology.

**Conclusions:**

The overall impact of these aging concepts on reshaping negative aging narratives remains relatively limited. Future efforts should focus on advancing technology, optimizing policies, enhancing social support systems, and fostering global collaboration to provide innovative and sustainable solutions that promote the overall well-being of older adults.

## Introduction

The global trend of an aging population is accelerating, with a steadily increasing proportion of people older than 65 years of age worldwide [1,2]. Many governments view this demographic change as an increasingly pressing challenge, particularly in the areas of health and social security [3]. Policy makers commonly associate aging with higher incidences of chronic diseases and increased reliance on long-term care services, which places significant burdens on public health budgets and resource allocation [4-7]. Similarly, many researchers view aging as a growing challenge, emphasizing how the prevalence of neurodegenerative diseases, diabetes, and other chronic conditions in older populations significantly increases medical demands, thereby intensifying pressure on health care systems [8-11].

Governmental focus on fiscal sustainability, along with academia’s emphasis on the challenges of the aging process, has fostered a prevailing negative narrative of aging as an inevitable social and economic burden. Policy documents frequently underscore the financial pressure aging places on pension systems and long-term care insurance, driving related legislation and measures [12]. This “cost-centric” perspective is also prevalent and echoed in academic studies, which highlight the strain aging poses to the labor market and its potential threat to national economic productivity [13-15].

In response to the prevalent focus on the negative impacts of aging, some researchers and international organizations (eg, World Health Organization [WHO]) have sought to reshape this narrative by introducing aging concepts like “healthy aging,” “successful aging,” and “active aging” [16]. These concepts seek to shift the focus from the diseases and functional decline associated with aging to the positive aspects of fostering holistic well-being and active social participation among older adults through preventive and supportive measures [17-21]. For example, the concept of active aging highlights the importance of continued participation in social, economic, cultural, and physical activities for older adults, and it emphasizes staying active in all areas of life to improve both the quality of life and overall health [22]. These concepts collectively aim to counteract the dominant “cost-centric” and deficit-based narratives of aging, promoting a more holistic view of older adults as active, valuable members of society.

However, despite the initial intentions behind these aging concepts, whether or not they have succeeded in counteracting the dominant negative narrative around aging remains largely unknown. In fact, some researchers even argue that these concepts may inadvertently reinforce certain aspects of the negative view of aging [23-25]. For example, Waddell et al [26] mentioned that achieving “successful aging” often emphasizes individual responsibility for maintaining health and well-being, overlooking broader social and environmental factors, setting unrealistic standards that marginalize those with health challenges, thus reinforcing a deficit-based view, and framing aging as a problem to be managed rather than a natural process. Additionally, the commercialization of “active aging” and “healthy aging” (eg, the “silver market” and “silver economy”) reinforces the notion that aging requires continuous self-improvement, perpetuating ageist views that devalue individuals who do not meet ideals of productivity or health [27,28].

Moreover, the definitions and applications of these aging concepts often vary significantly, leading to confusion and misinterpretation in both academic and practical contexts. For instance, “successful aging” from biomedical research is commonly defined as the absence of major diseases and the maintenance of good physical function in older people [29]. In contrast, successful aging in sociological and psychological studies emphasizes the ability of older adults to maintain social connections and mental well-being [30]. These definitional inconsistencies hinder cross-disciplinary understanding and cooperation and introduce uncertainties into the development and implementation of public health strategies and policy implementation.

Therefore, this study aims to examine whether, and in what ways, the application of aging concepts in academic research has contributed to reframing the dominant negative narrative surrounding aging. To achieve this, a systematic review of aging-related literature was conducted, leading to the identification of 16 key aging concepts. These concepts were then used as keywords to retrieve relevant papers from the Scopus database. Scopus’s built-in analysis tools, along with the bibliometric software VosViewer (Center for Science and Technology Studies), were used to map the distribution of publications across disciplines and journals, and to visualize international research collaborations and relationships between frequently cocited journals.

Furthermore, the linguistic software AntConc (Waseda University) supported a comprehensive content analysis, examining core themes, practical applications, persistent challenges, and emerging trends associated with these aging concepts. Based on these analyses, the study not only charts how aging concepts are defined, applied, and interpreted within academic discourse but also evaluates whether their application signals a shift from deficit-based to empowerment-oriented narratives.

To support this interpretive approach, the study draws on narrative theory (eg, [31]), which emphasizes how language and discourse shape the meanings ascribed to aging. Complementing this, the social construction of aging framework (eg, [32]) informs our understanding of aging concepts as dynamic constructs that are embedded in broader cultural, institutional, and policy contexts. Together, these theoretical models guide our analysis of how aging concepts contribute to either deficit-based or empowerment-oriented representations of later life.

## Methods

### Data Collection

This study adopts a systematic literature review approach, aiming to ensure the comprehensiveness and credibility of data through extensive searching, iterative reading, filtering, and cross-validation. The process began with the use of Google Scholar to identify aging concepts through close reading of academic journal papers, books, and conference proceedings. This initial stage was further enhanced by including authoritative documents from international organizations such as the WHO, the European Commission, and relevant government reports. This multilayered approach enabled the identification of 16 aging concepts as follows: (1) healthy aging, (2) successful aging, (3) active aging, (4) positive aging, (5) productive aging, (6) optimal aging, (7) social aging, (8) creative aging, (9) smart aging, (10) healthy-active aging, (11) adaptive aging, (12) robust aging, (13) resilient aging, (14) conscious aging, (15) intelligent aging, and (16) vital aging.

These 16 aging concepts were then used as keywords to search in the Scopus database. Scopus was selected for its extensive multidisciplinary coverage, inclusion of high-quality peer-reviewed journals across both biomedical and social sciences, and compatibility with bibliometric tools such as VosViewer. Its structured metadata and citation indexing made it especially suitable for large-scale quantitative and linguistic analysis. While Scopus provides a robust foundation for this study, future research may benefit from incorporating other databases (eg, Web of Science and PubMed) for triangulation and broader validation.

Initial searches occasionally returned irrelevant results, such as papers focused on animal aging or the degradation of materials and mechanical systems. To refine the dataset and focus on human aging, population-specific terms were added. The final search string was:

TITLE-ABS-KEY(“healthy aging” OR “active aging” OR “healthy-active aging” OR “smart aging” OR “intelligent aging” OR “successful aging” OR “creative aging” OR “social aging” OR “conscious aging” OR “resilient aging” OR “adaptive aging” OR “positive aging” OR “productive aging” OR “robust aging” OR “optimal aging” OR “vital aging”)AND(“elderly” OR “elderly people” OR “elders” OR “the aged” OR “the elderly population” OR “older adults” OR “older individuals” OR “older persons” OR “older generation” OR “senior citizens” OR “senior population” OR “seniors” OR “golden-agers” OR “retirees” OR “pensioners” OR “aging population” OR “aging adults” OR “geriatric population”)

Both American (“aging”) and British (“ageing”) spellings were used to ensure comprehensive retrieval. For compound terms such as “healthy-active aging,” both hyphenated and nonhyphenated forms were included. As of October 21, 2024, the final search yielded 10,642 papers in English-language peer-reviewed journals.

### Data Analysis

A combination of bibliometric, corpus linguistic, and qualitative content analysis methods was used to examine how aging concepts are applied and framed in academic literature. The built-in analytical tools of the Scopus database were used to perform an initial descriptive analysis of the dataset, including (1) the distribution of publications across various journals, (2) the trends in the number of publications over the years, (3) the spread of research across various academic disciplines, (4) and the emergence time of 16 aging concepts.

Following this, the bibliometric software VosViewer was used to visualize the co-occurrence of (1) geographic collaboration among countries, and (2) cocited journal references. The goal was to identify key regions contributing significantly to aging research and highlight highly influential journals. VosViewer is widely used for bibliometric analysis and scientific knowledge mapping, known for its exceptional capacity to manage large datasets, such as the 10,642 papers retrieved in this study. These network analyses offer insight into the structural dynamics of academic discourse, including disciplinary concentration and geographic dominance, which serve as contextual indicators for understanding the breadth and inclusivity of narrative development around aging concepts. In both cocitation and collaboration networks, total link strength (TLS) was used as a metric to quantify the strength of connections between nodes (eg, journals or countries). TLS is automatically calculated by VosViewer based on the frequency and intensity of co-occurrence—cocitation in journal networks, or coauthorship in country networks. A higher TLS value indicates stronger linkage and greater structural centrality within the network.

For the comparative analysis, 2 datasets were extracted:

- Dataset 1 consisted of a full set of 10,642 papers based on the 16 aging concepts, with extracted text from titles, abstracts, and keywords.
- Dataset 2 consisted of a focused set of 1402 papers retrieved using the keywords “age well,” “aging well,” and “ageing well,” also limited to titles, abstracts, and keywords.

The first dataset was concept-driven, focusing on how predefined aging concepts are applied in scholarly research, while the second dataset was goal-oriented, capturing how the broader notion of “aging well” naturally emerges in academic discourse. The second dataset was provided as a comparative lens, enabling an assessment of whether and how the selected aging concepts were embedded within mainstream narratives surrounding positive aging outcomes.

Then, linguistic analysis was performed using the software AntConc. Word frequency counts identified how often each aging concept appeared in both datasets, providing a basis for comparing their relative prominence. AntConc’s Keywords in Context function supported in-depth qualitative content analysis by extracting 30-word windows surrounding each concept for further analysis. Specifically, the initial step involved analyzing text from Dataset 1, which formed the foundation for subsequent analysis. The Keywords in Context function was then used to locate occurrences of the aging concepts within the text, extracting 30 words before and after each keyword to form target sentences. In the first round of content analysis, these target sentences were categorized into several main themes. The primary themes included:

- Core content: describes the main ideas of each aging concept.
- Application areas: refer to the domains where these concepts have been applied, such as public health or social policy.
- Challenges: highlight the difficulties encountered in achieving the goals of these aging concepts.
- Future trends: focus on the development directions and potential applications of these concepts.

**Figure 1.**
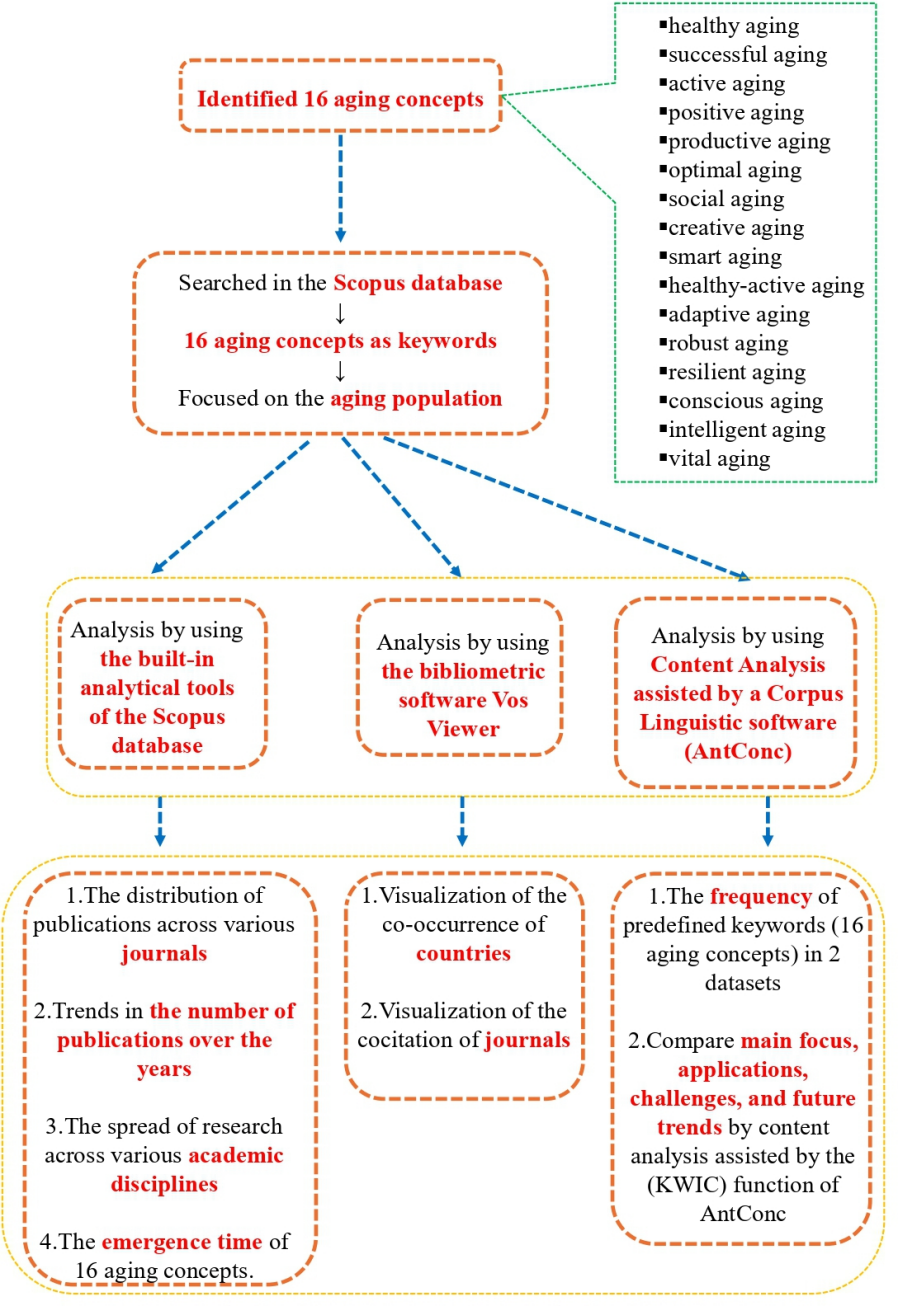
Research procedure. KWIC: Keywords in Context.

After completing the initial analysis, a second round of content analysis was conducted. In this phase, differences between the concepts were compared to provide a more summarized description—a “big picture,” so to speak. The process followed a clear and logical flow: beginning with large-scale text extraction, identifying keyword contexts, conducting 2 rounds of thematic content analysis, and concluding with a comparative synthesis. The analytical procedures are illustrated in Figure 1.

To evaluate whether aging concepts have contributed to shifting the negative narrative on aging, the content analysis examined the presence and emphasis of themes reflecting either deficit-based or empowerment-based narratives. Concepts framed primarily around disease, burden, and decline were interpreted as maintaining or even reinforcing a traditional negative discourse, while those emphasizing well-being, participation, adaptability, and holistic development were viewed as indicative of a shifting, more positive narrative change. The distribution of disciplines and publication trends also served as supporting indicators: a concentration of research within medical domains tended to reinforce traditional narratives, while an increasing presence in social, policy, or interdisciplinary domains suggested progress toward a more balanced narrative. These interpretive criteria guided the qualitative assessment of whether and how narrative transformation is occurring in the academic literature.

## Results

### Publication Trends and Disciplinary Distribution

#### Distributions of Publication Volume in Years

**Figure 2.**
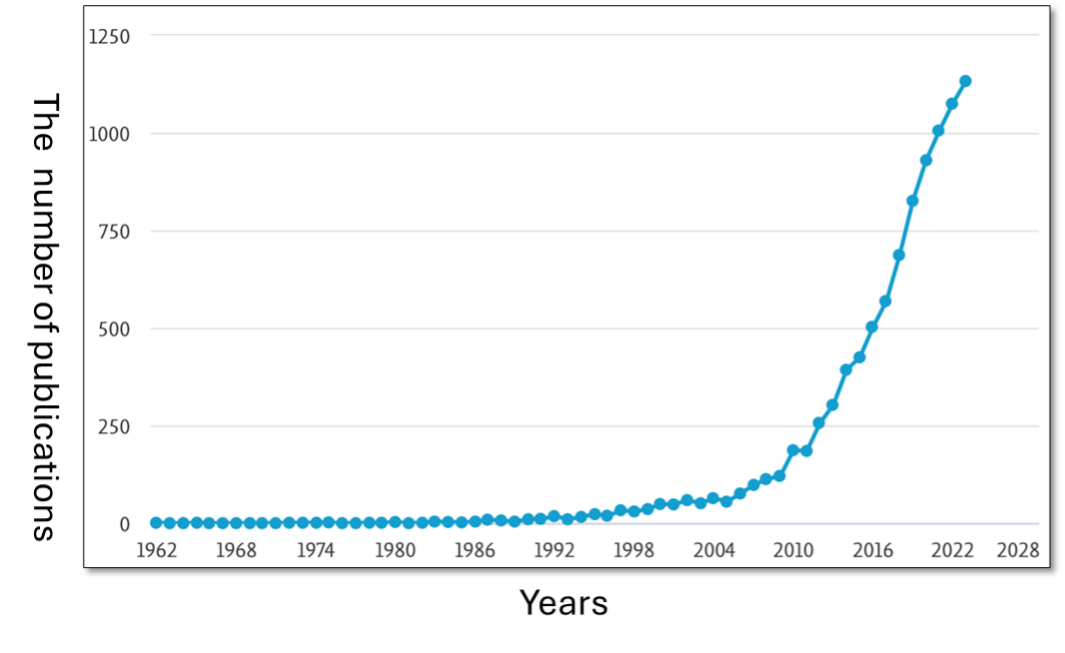
Trends in publications mentioning aging concepts (2016-2023).

The line chart (Figure 2) illustrates the annual publication volume trend over the 62-year period from 1962 to 2023, based on 9465 papers out of the 10,642 retrieved, as data for 2024 represents only part of the year, which affects the trend line.

It is clearly shown that there is an exponential increase in the number of papers published, particularly after 2011, which indicates a growing interest and emphasis on research mentioning aging concepts. In the early 50 years (from 1962 to 2011), the number of publications remained steady, but slow growth, generally below 250 per year. Then, in the near-decade (from 2012 to 2020), the number of publications experienced a sharp increase, with the count reaching between 250 and 1000 papers. This upward trajectory continued between 2021 and 2023, when the number of publications exceeded 1000 papers.

#### Distributions Cross Disciplines

The disciplinary distribution of publications is presented in Multimedia Appendix 1. Medicine takes the lead with 34.2%, clearly positioning it as the central discipline in research applying aging concepts. Social Sciences (12.4%) and Biochemistry and Genetics (9.8%) follow at a distance, indicating that the research disciplines extend beyond medicine to encompass the social sciences and biosciences. In contrast, Health Professions account for 3%, Environmental Science for 2.8%, and Computer Sciences for only 1.5%, indicating that these fields contribute fewer aging-related publications in absolute terms.

This distribution highlights an important structural feature of aging research: while it remains heavily concentrated in medical domains—often reflecting deficit-based narratives centered on disease, decline, and dependency—there is a gradual increase in contributions from disciplines that approach aging from more social, psychological, or interdisciplinary perspectives. This expansion suggests a modest shift toward a more multidimensional, empowerment-oriented discourse. However, it is worth noting that this analysis does not account for the total number of publications within each discipline. Therefore, it cannot measure the relative importance of aging within each field. Future studies could address this limitation by normalizing the data against overall publication volume within each discipline.

#### Distribution of Journals

Table 1 presents the distribution of the top 35 journals and the number of papers published in each (all with over 50 publications). This distribution reflects the broad scope and complexity across areas of research applying aging concepts, spanning fields such as public health, geriatrics, neuroscience, neurodegenerative diseases, aging psychology and mental health, sociology, social sciences, nutrition, and multidisciplinary aging studies. The top 5 journals in this list are the *International Journal of Environmental Research and Public Health*, with 322 papers; *BMC Geriatrics*, with 204 papers; *PLOS One*, with 148 papers*; Aging and Society,* with 144 papers*;* and *Frontiers in Aging Neuroscience,* with 142 papers.

**Table 1.** Top 35 journals ranking by the number of papers (as of October 21, 2024).

No.	Journal name	Papers, n
1	*International Journal of Environmental Research and Public Health*	322
2	*BMC Geriatrics*	204
3	*PLOS One*	148
4	*Aging and Society*	144
5	*Frontiers in Aging Neuroscience*	142
6	*Journals of Gerontology Series A: Biological Sciences and Medical Sciences*	140
7	*Neurobiology of Aging*	133
8	*Journals of Gerontology Series B: Psychological Sciences and Social Sciences*	128
9	*The Gerontologist*	103
10	*Frontiers in Public Health*	99
11	*Educational Gerontology*	95
12	*Experimental Gerontology*	91
13	*Nutrients*	87
14	*Aging and Mental Health*	86
15	*Archives of Gerontology and Geriatrics*	86
16	*BMC Public Health*	86
17	*Psychology and Aging*	86
18	*NeuroImage*	85
19	*Aging Clinical and Experimental Research*	77
20	*Journal of the American Geriatrics Society*	73
21	*Journal of Nutrition, Health and Aging*	72
22	*Scientific Reports*	71
23	*Journal of Aging and Social Policy*	62
24	*Journal of Alzheimer’s Disease*	62
25	*Social Science and Medicine*	62
26	*Age and Aging*	61
27	*Journal of Aging and Health*	61
28	*BMJ Open*	60
29	*Frontiers in Psychology*	57
30	*Journal of Applied Gerontology*	56
31	*Gerontology*	53
32	*Aging International*	52
33	*Journal of Aging Studies*	52
34	*Activities, Adaptation and Aging*	51
35	*Aging*	50

#### Co-Occurrence of Cocited Journals

**Figure 3.**
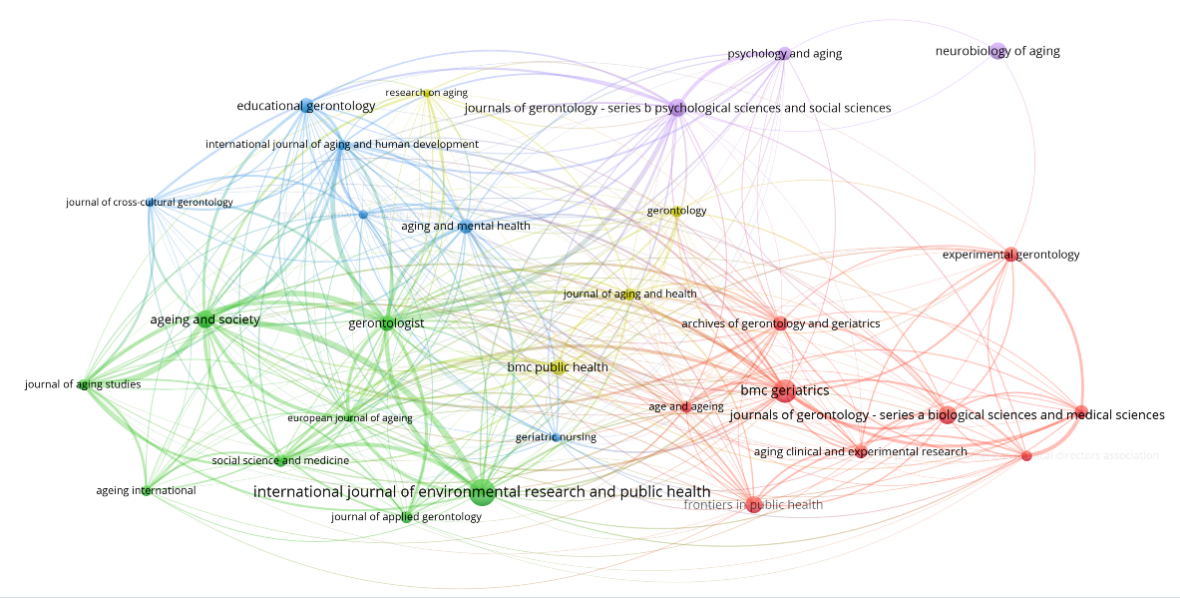
The map of the cocitation network of journals generated by VosViewer.

Figure 3 presents a cocitation map of journals in research applying aging concepts generated using VosViewer software. Table 2 lists the top 30 journals ranked by the number of citations. Together, they show key journals’ academic influence and interconnections.

Specifically, larger nodes, such as *Journals of Gerontology—Series A: Biological Sciences and Medical Sciences* (6981 citations), *Gerontologist* (6774 citations), *Journals of Gerontology—Series B: Psychological Sciences and Social Sciences* (5016 citations), and *Aging and Society* (4784 citations), indicate that these journals hold considerable academic influence in research applying aging concepts. In addition to the node size, different colors in the network distinguish various research domains, such as social sciences and public health (green), clinical and biological aging (red), cognitive aging and neuroscience (purple), educational and developmental aspects of aging (blue), and a multidisciplinary approach combining medical and social sciences (yellow). Moreover, the thickness of the connecting lines between journals reflects the strength of their citation relationships, quantified by TLS, which indicates how frequently the journals are cocited. The top 3 journals in terms of TLS are *Gerontologist* (TLS*:* 345), *Aging and Society* (TLS: 317), *and International Journal of Environmental Research and Public Health* (TLS: 230), highlighting strong citation linkages within the research network. As a cumulative metric automatically calculated by VosViewer, TLS represents the total strength of cocitation links a journal has with others. A higher TLS means the journal is frequently and widely cocited, suggesting its centrality in the knowledge network. This clustering of cocitation relationships, dominated by biomedical and gerontology-focused journals, reflects the structural concentration of aging discourse, indicating a limited narrative shift as defined in our methodological framework.

**Table 2.** Cocitations of top 30 journals: ranked by TLS^a^.

No.	Journals	Citations, n	TLS
1	*Gerontologist*	6774	345
2	*Aging and Society*	4784	317
3	*International Journal of Environmental Research and Public Health*	3646	230
4	*International Journal of Aging and Human Development*	1544	193
5	*BMC Geriatrics*	3397	183
6	*Social Science and Medicine*	3063	173
7	*Aging and Mental Health*	2526	171
8	*Journals of Gerontology—Series B: Psychological Sciences and Social Sciences*	5016	161
9	*Journal of Aging Studies*	2323	153
10	*European Journal of Aging*	793	145
11	*Archives of Gerontology and Geriatrics*	1763	128
12	*American Journal of Geriatric Psychiatry*	3017	123
13	*Educational Gerontology*	1377	123
14	*Journal of Aging and Health*	1861	112
15	*Journals of Gerontology—Series A: Biological Sciences and Medical Sciences*	6981	108
16	*Journal of Nutrition, Health and Aging*	1858	99
17	*Age and Aging*	1958	93
18	*Journal of Applied Gerontology*	749	91
19	*Journal of Cross-Cultural Gerontology*	725	91
20	*Psychology and Aging*	4752	90
21	*Frontiers in Public Health*	654	87
22	*Journal of the American Medical Directors Association*	2411	82
23	*BMC Public Health*	1974	81
24	*Research on Aging*	1052	80
25	*Aging Clinical and Experimental Research*	1273	79
26	*Geriatric Nursing*	882	79
27	*Aging International*	720	72
28	*Experimental Gerontology*	2890	70
29	*Geriatrics and Gerontology International*	461	64
30	*Gerontology*	1675	64

a TLS: total link strength.

#### Distributions in Geographical Areas

**Figure 4.**
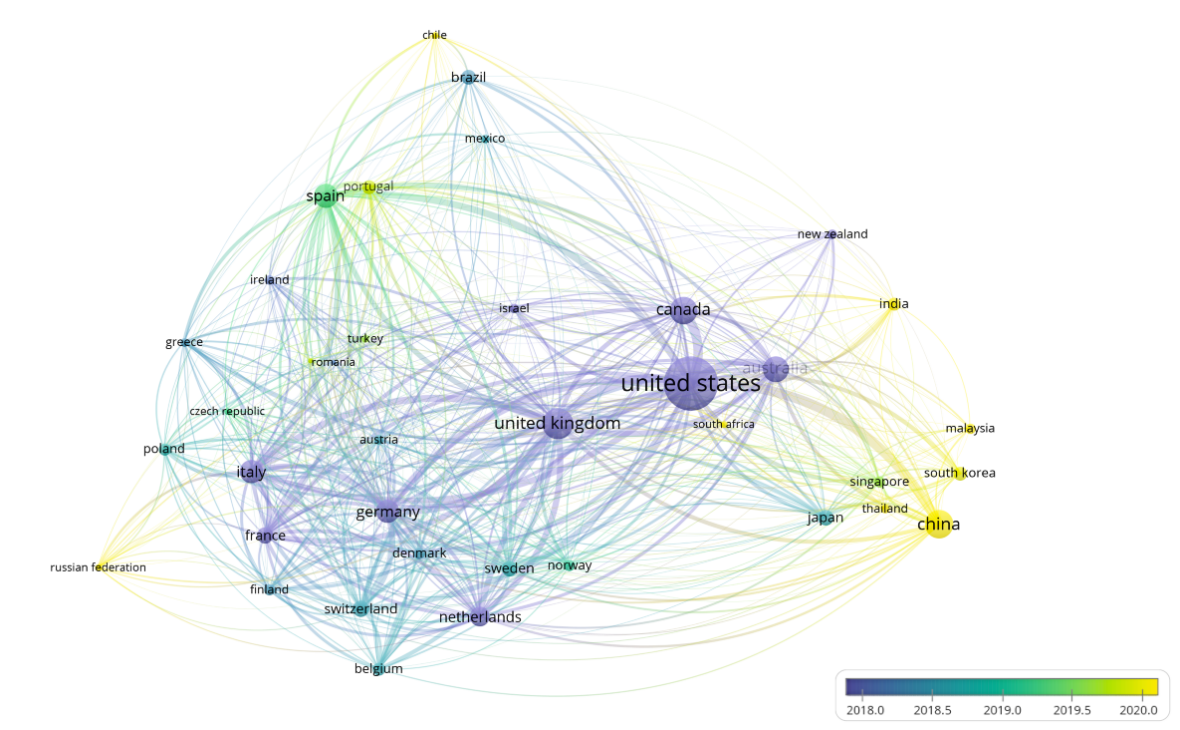
Global collaboration network map generated by VosViewer.

Figure 4 presents a collaboration map of countries generated by VOSviewer, while the number of papers ranked and the TLS for each country are listed in Table 3. These 2 tables provide a comprehensive overview of global collaboration in research applying aging concepts.

The countries such as the United States, China, and the United Kingdom play leading roles in the global collaboration network. Specifically, the size of the nodes reflects each country’s importance in the network, with larger nodes indicating a higher number of published papers. The United States leads with 3262 papers, followed by China with 1447 papers, and the United Kingdom with 1094 papers (Table 3). The lines connecting the nodes represent the collaboration between countries, where thicker lines signify stronger collaborative relationships. This strength is quantified by the TLS, as shown in Table 3. In this regard, the United States, the United Kingdom, and China rank the highest in TLS as well, with values of 1792, 1425, and 779, respectively. In this case, TLS reflects the cumulative strength of coauthorship ties a country maintains with others, helping identify central players in the global landscape of aging research collaboration.

Additionally, in earlier years (eg, around 2017), collaborations were predominantly centered in Western countries such as the United States, Italy, Germany, France, Switzerland, and the Netherlands, as indicated by the blue and purple lines in Figure 4. In contrast, more recent collaborations (eg, around May 2019) are represented by green and yellow lines, showing a growing focus on research applying aging concepts in Asian countries, with China leading the way. This shift has also seen increased collaborations involving countries like South Korea, Thailand, Malaysia, and Singapore, reflecting a broadening of the global research network. Nevertheless, the persistent dominance of high-income Western countries in collaborative centrality suggests an imbalanced global discourse, potentially constraining the diversification of narrative perspectives in aging research.

**Table 3.** Top 30 countries: ranked by the number of papers.

No.	Country	Papers, n	Total link strength
1	United States	3262	1792
2	China	1447	779
3	United Kingdom	1094	1425
4	Canada	871	595
5	Australia	776	726
6	Spain	664	800
7	Italy	658	944
8	Germany	635	871
9	Netherlands	464	661
10	France	338	624
11	Switzerland	325	638
12	Sweden	298	532
13	Japan	282	279
14	Brazil	259	175
15	Portugal	242	313
16	South Korea	220	151
17	Belgium	206	375
18	India	200	141
19	Poland	178	261
20	Singapore	168	216
21	Denmark	161	300
22	Norway	160	251
23	Finland	151	324
24	Malaysia	132	82
25	Israel	124	175
26	Ireland	118	185
27	Austria	117	302
28	New Zealand	106	86
29	Greece	103	314
30	Thailand	101	87

### Analysis of Aging Concepts: Frequencies, Emergence, and Categorization

#### Frequencies of 16 Aging Concepts in Aging-Related Research

Multimedia Appendix 2 presents the frequency of aging concepts identified through the corpus linguistic software AntConc, analyzing titles, abstracts, and keywords across 10,642 papers (dataset 1). These frequency data reflect the academic attention given to different aging concepts, and higher frequencies suggest greater scholarly interest, while lower frequencies may indicate emerging or more niche research areas.

The most frequently used aging concept is “healthy aging,” which appears 9710 times and indicates that health is a major focus in aging-related research. Other commonly used aging concepts include “successful aging” (5237 times) and “active aging” (3210 times), reflecting attention on maintaining function and engagement in older age. On the other hand, less frequently mentioned concepts are “robust aging” (8 times) and “conscious aging” (17 times), suggesting that these concepts are less explored or emerging in aging-related research.

#### Emergence Time of 16 Aging Concepts

**Figure 5.**
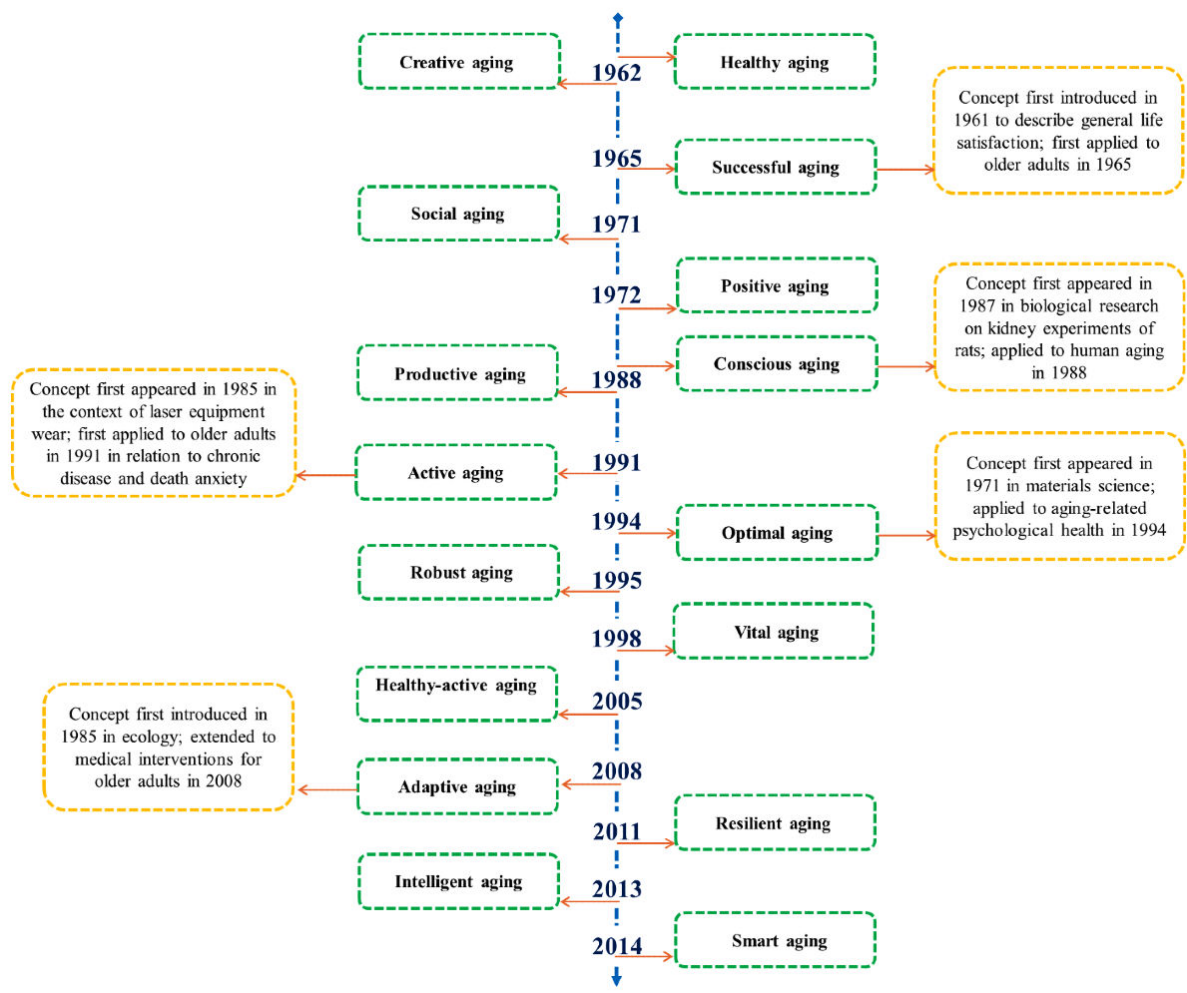
Timeline of aging concepts first applied to older adult populations in academic research. The main timeline indicates the year when each concept was first applied in academic research, focusing specifically on human aging or older adults. Additional dates in red indicate when the concept first appeared in academic literature, including in nonaging or nonhuman contexts such as materials science, ecology, or animal research.

Figure 5 presents the years when aging concepts were first explicitly applied to older adult populations in academic literature, revealing their increasing diversity over time. Notably, to explore the differences in the initial emergence time and application of these concepts, a comparison was made between the search results of 16 aging concepts when used as standalone keywords and when combined with keywords related to the aging population. In Figure 5, the emergence time of each aging concept reflects the first documented use in academic literature where the term is explicitly applied to human aging or older adults, rather than its first-ever mention in any unrelated scientific field.

In terms of the time dimension, the earliest aging concepts are “healthy aging” (1962), “creative aging” (1962), and “successful aging” (1965). These concepts were initially introduced from nutrition, gerontology, and medicine perspectives. The first concept was “healthy aging,” which originated from a nutritional perspective, focusing on how nutrition can slow down physical decline and prevent diseases to improve the quality of life for older adults [33]. “Creative aging” initially encouraged reflection on the reasons behind the pursuit of longevity, emphasizing that beyond disease management, attention should be given to personal growth and the richness of life experiences [34,35]. The concept of “successful aging” first appeared in 1961 in general psychological literature, but its first explicit use in aging-related research occurred in 1965 [36]. Research in 1965, focusing on the facts of impacting aging for older adults, highlighted the need to distinguish between aspects of aging due to the natural aging process and those more closely related to individual lifestyles or situational factors unrelated to aging [37].

From the 1970s to the 2000s, perspectives on aging concepts gradually expanded, introducing a range of ideas, including social aging in 1971, optimal aging in 1994, positive aging in 1972, adaptive aging in 2008, active aging in 1991, conscious aging in 1988, productive aging in 1988, robust aging in 1995, and vital aging in 1998. Specifically, in 1971, the concept of “social aging” was introduced for the first time, emphasizing that the irreversible loss of an individual’s ability to choose what they can do is the most important indicator of aging, whether social or physical aging [38]. That same year, the concept of “optimal aging” also emerged, initially referring to the aging process of metals [39]. It was not until 1994 that the term was applied to older adults, proposing that a positive mental outlook could help them cope constructively with the challenges of aging [40]. In 1972, the term “positive aging” appeared for the first time in the literature, mainly in relation to controlling Hodgkin’s disease [41]. In 1985, “adaptive aging” was first mentioned in the field of ecology [42], and by 2008, the concept had been applied in medical research related to older adults [43]. The term “active aging” initially appeared in the literature related to laser equipment wear [44], but was first applied to human aging in 1991, when discussions on chronic disease and death anxiety associated the term with older adults [45]. In 1987, the term “conscious aging” first appeared in biological research related to kidney experiments on rats [46]. The year 1988 is used in the timeline to reflect the earliest application of the term to human aging. In 1988, the concept of “productive aging” emerged, which highlighted how aging amplified economic, political, and moral issues, shifting the focus toward social policy [47]. “Robust aging” was first introduced in 1995, with criteria including greater social interaction, better health and vision, and fewer significant life events for older individuals [48]. Finally, in 1998, the concept of “vital aging” was introduced in psychological health programs, focusing on the development of mental health skills and the effectiveness of changing attitudes, beliefs, and behaviors, providing the motivation and support needed for individuals to make important lifestyle changes and create a more vibrant and productive later life [49].

In the 21st century, concepts like “healthy-active aging” (2005) and “resilient aging” (2011) were introduced, and with the rapid advancement of technology, newer concepts such as “intelligent aging” (2013) and “smart aging” (2014) also emerged. In particular, “healthy-active aging” was first introduced in 2005, suggesting that free health checks and financial advice can be effective for older adults, while knowledge acquisition and social activities enhance their sense of control, especially in impoverished areas [50]. The concept of “resilient aging” was introduced in 2011, offering a useful framework for understanding the mechanisms of aging in the context of disease-related pathologies [51]. “Intelligent aging” emerged in 2013 in response to the growing aging population and shrinking tax base, increasing demand for health care and housing services, while IAServ, a cloud-based platform, aims to provide personalized, scalable, and cost-effective home care through modern technology [52]. In 2014, the concept of “smart aging” was first introduced, emphasizing a positive acceptance of later life and framing aging as a developmental stage toward intellectual maturity [53].

In summary, the years presented in Figure 5 are selected based on a consistent criterion: only the first documented applications explicitly related to human aging or older adult populations are included. Aging concepts have evolved from a narrow focus on health and success to encompass a broader, multidimensional view that includes psychological, social, and technological aspects. Over time, society’s understanding of aging has deepened, and future research in this field will likely emphasize interdisciplinary approaches to address the complex challenges of aging. This shift in perspective not only reflects changing societal needs but also provides more comprehensive strategies for dealing with the aging population.

#### Top 3 Aging Concepts: Publication Growth and Trends Comparison

**Figure 6.**
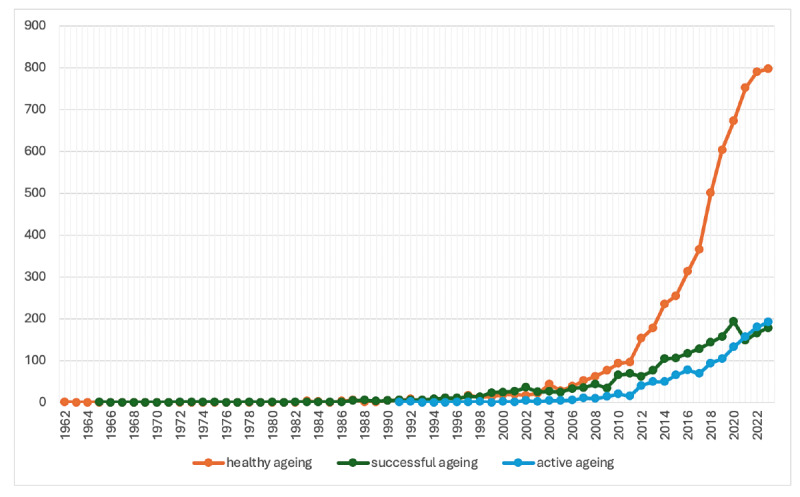
Successful aging, healthy aging, and active aging concepts: publication trends over time (1961-2023). Frequency of 16 aging concepts in achieving “age well.”

Figure 6 shows the top 3 popular aging concepts among the 16 identified: “healthy aging,” “successful aging,” and “active aging,” reflecting significant growth from 1962 to 2023, but at varying rates. “Healthy aging” saw a sharp rise in attention after 2010, with a particularly rapid increase after 2020, becoming the dominant aging concept mentioned in research. On the other hand, “successful aging” and “active aging” gained more steady attention starting from the 1990s. “Active aging” experienced slightly faster growth after 2000, and especially after 2019, surpassing “successful aging” in growth rate, reflecting an increased emphasis on older adults’ participation in social activities and maintaining an active lifestyle.

This trend suggests that while “healthy aging” has become the dominant concept, the steady growth of “active aging,” particularly its acceleration after 2019, highlights an increasing emphasis on the importance of older adults actively participating in society and maintaining an engaged lifestyle. Compared to other more established concepts, there is likely to be a greater future focus on leveraging “active aging” to improve the quality of life for older adults, encouraging their active social integration and promoting their overall well-being.

Multimedia Appendix 3 shows the frequency of 16 aging concepts in the text of titles, abstracts, and keywords across 1402 papers (dataset 2: searched in the Scopus database using the keywords “age well” OR “aging well” OR “ageing well” limited to English-language journal papers), identified through the corpus linguistic software AntConc. This comparison evaluates how effectively the identified aging concepts from dataset 1 are reflected in the more naturalistic academic discourse surrounding “aging well,” thereby indicating their narrative integration and relevance across different contexts. The comparison of the use frequency of different aging concepts reveals that “successful aging (417 times)” is the popular aging concept in discussions of how to achieve “age well,” followed closely by “healthy aging (247 times)” and “active aging (180 times).”

Additionally, less frequently mentioned concepts, such as “positive aging” and “productive aging,” are mentioned 70 and 14 times, indicating that while less prominent, there is still interest in aging’s psychological and social contribution aspects. Aging concepts such as “adaptive aging” and “resilient aging” appear very rarely, with just 3 and 1 occurrences, suggesting that these concepts, though relevant to coping with aging challenges, have not yet gained significant traction in the literature. Likewise, concepts like “intelligent aging” and “smart aging” were not found, reflecting that while there may be much research into how technology supports older adults in adapting to their environments, they have yet to become mainstream but may hold potential for future exploration.

#### Categorization and Core Focus of 16 Aging Concepts

Table 4 summarizes 16 aging concepts, detailing their core focus and application areas and categorizing them into 4 groups, including (1) health and well-being, (2) social engagement, (3) technology and innovation, and (4) resilience and adaptation. Analyzing these concepts reveals how the academic community prioritizes different aspects of aging and applies them in practice. Although all the concepts share the common goal of improving the quality of life for older adults, each contributes a distinct perspective to the field of aging-related research.

The concepts of aging, such as healthy aging, successful aging, optimal aging, positive aging, and vital aging, can be categorized as part of the Health and Well-being Aspect. These concepts aim to promote health and overall well-being, yet they approach this goal from varying angles. Healthy aging primarily promotes overall health through public health initiatives and disease prevention. Successful aging highlights the importance of maintaining both physical and mental health, focusing on how psychological well-being and social support enhance quality of life. Optimal aging takes a broader approach, addressing physical, emotional, and cognitive health, aiming to extend a healthy lifespan through community well-being and health promotion programs. Positive aging focuses more on mental well-being, such as maintaining a positive attitude and emotional resilience. Finally, vital aging stresses lifelong adaptation for their well-being, encouraging older adults to remain independent and active through lifelong learning and adaptability.

**Table 4.** Comparisons of the core focus of 16 aging-related concepts.

Categorizations and aging concepts		Core focus	Application areas
Health and well-being aspect			
Healthy aging		Focuses on achieving a high quality of life through promoting overall health and preventing disease during the aging process.	Public health Older people care Disease prevention
Successful aging		Focuses on maintaining physical and mental health in later life and enhancing quality of life.	Older people health management Mental health Social policy
Optimal aging		Focuses on promoting health, longevity, and overall well-being by addressing physical, emotional, and cognitive aspects of aging, ensuring individuals remain independent and active as they age.	Health promotion Community well-being Emotional and sensory wellness Public health programs Evidence-based information Dissemination
Positive aging		Focuses on the importance of maintaining a positive attitude, psychological resilience, and emotional well-being.	Mental health Emotional well-being Psychological therapy
Vital aging		Emphasizes lifelong adaptation to maximize health, independence, and cognitive and emotional well-being, promoting active and successful aging through various programs and interventions.	Lifelong adaptation Mental fitness Lifelong learning Environmental policies Cultural context Community-based programs
Creative aging		Focuses on enhancing the psychological and social well-being of older adults through artistic and cultural activities.	Art or culture engagement Psychological therapy
Social engagement aspect			
Active aging		Focuses on the active participation of older adults in social activities and maintaining physical vitality.	Social participation Community activities Sports and recreation
Social aging		Focuses on the roles and interactions of older adults in society, examining how social, economic, and cultural factors influence the aging process.	Sociological research Social policy Analysis of older adults’ social roles
Productive aging		Focuses on the active participation of older adults in socially productive activities, such as volunteering, caregiving, and work, contributing to society while enhancing individual well-being.	Community volunteering Social support Health literacy Cultural differences Government policies Workforce participation Lifelong learning
Conscious aging		Emphasizes deep reflection on the aging process, spiritual growth, and contemplation of life.	Spiritual requirements Lifelong learning Personal growth
Health and well-being and social engagement aspect			
Healthy-active aging		Combines elements of healthy aging and active aging to maintain vitality and health in older adults.	Health promotion Community health Older people care
Technology and innovation aspect			
Smart aging		Focuses on supporting the quality of life of older adults through smart technologies such as virtual reality, data privacy protection, and smart cities.	Smart homes Health monitoring Smart city development
Intelligent aging		Mainly involves the application of smart technology in health monitoring and management.	Smart health monitoring Personalized health care AI^a^-assisted living
Resilience and adaptation aspect			
Adaptive aging		Emphasizes optimizing design and infrastructure to accommodate the physical and cognitive changes associated with aging.	Public infrastructure design Product design Biomedical research
Resilient aging		Emphasizes the adaptability and psychological resilience of older adults in the face of challenges.	Mental health Coping strategies Crisis management
Robust aging		Focuses on maintaining vitality across physical, emotional, cognitive, and social domains, with an emphasis on resilience and adaptability throughout the aging process.	Productive involvement Affective status Functional status Cognitive status Physical activity Social engagement Cellular biomarkers Cross-generational aging

a AI: artificial intelligence.

In a related area, aging concepts of active aging, social aging, productive aging, creative aging, and conscious aging all fall under the aspect of social engagement, but they each emphasize different areas. Active aging encourages older adults to stay involved in social and physical activities, promoting vitality. Social aging examines the social roles of older adults and the impact of social, economic, and cultural factors. Productive aging highlights older adults’ contributions to society through activities like volunteering and work, enhancing well-being. Creative aging improves emotional health through engagement in art and culture, while conscious aging emphasizes personal and spiritual growth through reflection on life. Notably, healthy-active aging belongs to both healthy-active aging and social engagement aspects because it combines elements of both healthy and active lifestyles, supporting older adults in maintaining vitality and well-being by staying physically and mentally active.

The technology and innovation aspect encompasses aging concepts like smart aging and intelligent aging, both of which address the role of technology in supporting older adults. Although both concepts highlight the importance of smart technologies, they diverge in their specific applications. Smart aging focuses on enhancing daily life through technologies such as smart homes and health monitoring systems, while intelligent aging integrates artificial intelligence (AI) and data science to provide personalized health care solutions, illustrating the increasing role of AI in aging research. While both are grounded in technology-based theories, intelligent aging emphasizes more prominently data-driven approaches and AI.

Within the resilience and adaptation aspect, concepts like resilient aging, adaptive aging, and robust aging focus on older adults’ capacity to adjust to the changes accompanying aging. Resilient aging highlights psychological resilience, emphasizing older adults’ ability to cope with challenges. On the other hand, adaptive aging explores how design and infrastructure can be optimized to accommodate physical and cognitive changes in aging. Robust aging takes a more comprehensive approach, incorporating resilience across physical, cognitive, emotional, and social domains and integrating biological markers to assess longevity and health outcomes.

### Challenges and Future Trends of Achieving Aging Concepts

The 16 aging concepts reflect the academic community’s vision of promoting “aging well” from various perspectives. Through content analysis, Table 5 outlines the challenges in achieving the goal of each aging concept and future trends in applying each aging concept. Although the challenges—such as unequal access to resources, technological barriers, and cultural differences—are significant, there are promising opportunities for innovative and sustainable solutions. Advances in technology and a growing global focus on aging will create opportunities for policy makers, researchers, and communities to collaborate in addressing these challenges. By doing so, older adults worldwide can anticipate a safer, healthier, and more fulfilling aging process, regardless of their social, cultural, or economic backgrounds.

**Table 5.** Challenges and future work of 16 aging-related concepts.

Aging concepts	Challenges in achieving the goal of each aging concept	Future trends in applying each aging concept
Healthy aging	Lack of interdisciplinary collaboration and long-term investment Unequal access to medical resources globally	Greater integration of healthy aging initiatives in public health policies Growing focus on preventive health management due to accelerated global population aging
Successful aging	Challenges in defining “success” across diverse cultural backgrounds Difficulty in setting a universal standard due to significant variation in individual health status	Increasing emphasis on social policies aimed at supporting older adults to maintain a high quality of life in their later years Expanded community support, health care, and mental health services for older adults
Active aging	Challenges of physical and environmental barriers for sustained participation Difficulty for older adults in economically and infrastructurally disadvantaged areas	Advances in technology enabling digital activities and remote participation as new means for older adults to engage socially Increased participation of older adults in social activities through web-based communities and digital courses
Positive Aging	Challenges due to negative stereotypes of aging hindering broad acceptance Limited resources to address mental health issues in older adults across sectors	Increasing awareness of mental health leading to greater focus on the mental health and emotional well-being of older adults More mental health services and support programs emerging to address the needs of older adults
Smart aging	Barriers due to high costs of technology application and unresolved data privacy issues Challenges in older adults’ acceptance of technology	Further development and widespread adoption of smart homes, wearable devices, and health monitoring technologies Enhanced safety and independence for older adults living at home through advanced technologies
Social aging	Challenges arising from differences in social structure and culture for older adults Difficulty in developing universal solutions due to varying social challenges across countries and communities	Increased focus on aging issues leading to more policies and research on role transitions of older adults in society Emphasis on cultural and social support to enhance social participation of older adults
Creative aging	Insufficient resources for creative activities in certain areas Limited opportunities for older adults in remote or resource-scarce communities	Remote art programs and digital creative workshops providing opportunities for older adults to engage in creative activities when in-person participation is not possible Growing importance of digital platforms postpandemic facilitating creative engagement for older adults
Adaptive aging	Challenges due to the diversity and complexity of older adults’ needs for product design Significant time and financial resources required to promote and apply adaptive design in public infrastructure	Increasing focus on adaptive and accessible design in the construction of future cities and public infrastructure Ensuring safe and comfortable environments for older adults through improved urban and infrastructure design
Resilient aging	Challenges due to considerable variability in individual psychological resilience Imbalances in social support systems affecting older adults’ ability to build resilience across countries and communities	Fostering psychological resilience in older adults becoming a priority amid increasing global social and environmental crises Further development of psychological support programs and community support networks for older adults
Healthy-active aging	Challenges in coordinating resources across multiple fields for multidimensional health Difficulty in achieving health goals in economically underdeveloped or resource-limited regions	Expansion of community health programs, particularly through digital health platforms Promotion of healthy behaviors among older adults via digital health initiatives
Intelligent aging	Challenges due to high cost and complexity of technology limiting its application range Difficulty in acceptance of smart technologies by older adults	Increasing role of AI^a^ and big data in personalized health management Emergence of more AI-based health solutions for older adults
Conscious aging	Challenges due to varying cultural importance placed on the spiritual life of older adults Limited societal attention to spiritual health compared to physical health hindering development in this area	Increasing global attention to mental and spiritual health leading to greater promotion of spiritual support programs for older adults More widespread adoption of practices such as meditation and yoga among older adults
Productive aging	Challenges due to disparities in access to social support and education Varying cultural perspectives on aging as a barrier Unequal access to workforce participation opportunities for older adults	Increasing promotion of productive aging through policy initiatives, focusing on the integration of older adults in the community and workforce Key trends of lifelong learning and flexible work opportunities for older adults
Robust aging	Challenges due to variability of aging experiences across generations Disparities in access to health care and social support systems Differences in biomarker availability and interpretation	Advancements in biotechnology and understanding of cellular aging driving robust aging strategies toward personalized health interventions Importance of cross-generational health programs and adaptive aging practices in future public health initiatives
Optimal aging	Challenges due to gender disparities in aging experiences Unequal access to health care and wellness programs Need for broader dissemination of reliable health information	Growing emphasis on evidence-based health interventions and gender-specific aging strategies Increasing focus on optimal aging through personalized health plans, community support systems, and stress management techniques
Vital aging	Challenges due to disparities in educational opportunities Environmental inequalities impacting aging experiences Cultural differences in aging experiences across regions Accessibility issues of programs in different regions	Focus on expanding vital aging programs globally, emphasizing integration of environmental policies Attention to mental fitness initiatives to enhance aging experiences across different cultural contexts

a AI: artificial intelligence.

One of the primary challenges in achieving the goal of these aging concepts is the global disparity in medical and health care resources, particularly in economically disadvantaged regions where access to medical services is limited. This inequality hampers older adults’ ability to maintain both physical and mental health and benefit from preventive health care strategies. In the future, public health efforts should focus on integrating preventive health care with personalized interventions, ensuring that older adults have access to the necessary resources to support their well-being, regardless of location.

In addition to health care challenges, promoting social participation among older adults presents another major obstacle. Factors such as social structures, economic development, and cultural differences often create barriers to maintaining social connections and engaging with communities. Many societies prioritize physical health while neglecting emotional and spiritual needs. Cultural differences also influence the emphasis placed on spiritual life, making it difficult to implement universally applicable support systems. However, with growing global awareness of mental health, more programs are likely to address the emotional and spiritual well-being of older adults. Practices such as meditation, yoga, and other spiritual activities are expected to become more widespread, fostering a sense of fulfillment and psychological balance. Additionally, in many regions, older adults struggle to fully participate in social life due to a lack of flexible employment options and inadequate lifelong learning opportunities, further limiting their engagement. Balancing physical health with mental and spiritual well-being remains crucial. To improve the quality of life for older adults, future policies should focus on strengthening social support networks and providing meaningful opportunities for social participation, which in turn will enhance their mental health and sense of belonging.

Adopting technology to improve older adults’ lives also presents unique challenges. While technological solutions like smart homes, wearable devices, and health monitoring systems hold great potential, their high costs and complexity often limit widespread adoption, particularly in resource-constrained regions. Privacy concerns further complicate the integration of these technologies into everyday life. Future technological developments must focus on reducing costs, improving accessibility, and ensuring data security to make these tools more inclusive. Additionally, creative activities remain unevenly distributed, especially in remote areas, which are key to promoting older adults’ emotional and social well-being. Expanding web-based platforms will provide older adults with greater access to creative opportunities, helping bridge the gap between regions with varying resource availability.

Finally, fostering older adults’ adaptability to changing environments and promoting psychological resilience is crucial for their overall well-being. Developing these qualities requires significant time and financial investment. Public infrastructure and services must be optimized to meet the diverse needs of older adults, ensuring they can live in safe and comfortable environments. Building community support systems and mental health programs will also be critical in fostering psychological resilience, especially as global social and environmental crises increasingly affect vulnerable populations. Future urban planning efforts should prioritize adaptive design to create environments that support older adults’ physical, mental, and emotional health.

These challenges and future trends were derived from the content analysis process and thematically coded based on recurring contextual patterns. They also provide insight into the limitations of existing aging narratives and help identify conceptual gaps that could inform a more inclusive and multidimensional shift in aging discourse.

## Discussion

### Principal Findings

This study found that although a wide range of aging concepts have been introduced in academic literature, their overall impact on reframing negative aging narratives remains limited. The analysis revealed that most applications of these concepts continue to be concentrated in biomedical fields, with relatively less integration of social and economic dimensions. While some concepts promote multidimensional perspectives, their practical influence on shifting aging discourse is still fragmented and uneven. The demographic shift toward an aging population has led to rising concerns about “ageism,” which reinforces stereotypes of older adults as cognitively and physically declining, resistant to change, or burdensome to society [54,55]. These views contribute to social exclusion and negatively affect older adults’ mental and physical well-being. Academic research plays a key role in challenging such narratives by adopting a more comprehensive view of aging that encompasses physical, cognitive, emotional, social, and economic dimensions [56,57]. Recognizing older adults’ contributions—through work, caregiving, community participation, and cultural knowledge—can reshape how society perceives aging and promote age-inclusive policies [58-60].

The primary goal of this study is to explore whether and how research applying aging concepts contributes to shifting the negative narrative on aging in academia. The overall results show that while great efforts have been made, progress has been limited. As outlined in the Methods section, the dominance of biomedical framing and the lack of social and economic integration indicated a limited narrative shift. While the bibliometric analysis reveals a significant increase in aging concept research across disciplines, the concentration in medical fields and minimal attention to social and economic aspects still reflect an uneven narrative shift. Although many of these studies aim to promote “aging well,” they often take a narrow approach, focusing mainly on medical aspects such as disease control, health maintenance, and longevity. This limited perspective, while contributing to a partial change in the perception of aging, can inadvertently reinforce stereotypes (thinking: equating aging with poor health), prejudice (feeling: viewing older adults as less capable), and discrimination (acting: excluding older adults from work and daily life) [61]. Aging is a natural part of life and requires medical support across the human lifespan, but a holistic perspective on aging should extend beyond medical aspects to include social, economic, and other dimensions, generating a more balanced and comprehensive understanding of aging [62-64].

While the emergence of diverse aging concepts reflects an aspirational effort to counter negative narratives, our findings suggest that their actual impact has been uneven and often limited. Several factors may help explain this discrepancy. Many aging concepts lack consistent definitions, making them difficult to operationalize across disciplines and policy settings. The continued dominance of a biomedical focus further restricts their narrative potential. Furthermore, some concepts—such as “successful” or “active” aging—have been critiqued for reinforcing idealized standards or placing the responsibility of healthy aging on individuals, which may unintentionally perpetuate ageist assumptions. These limitations highlight the gap between conceptual intention and actual discursive impact.

These findings can be further interpreted through the lens of narrative theory and the social construction of aging, which suggests that aging concepts do not merely describe reality but actively shape how aging is perceived and valued in society. This theoretical perspective helps explain the persistence of deficit-based frames, and the limited extent of narrative change remains despite growing conceptual diversification.

At the same time, however, research involving these aging concepts has also acknowledged the multidimensional nature of aging and highlighted the need to address it holistically in both research and policy. The content analysis of this study categorized all 16 aging concepts into four groups based on their main focus and application, contributing to a more comprehensive understanding: (1) health and well-being (eg, healthy aging, successful aging, optimal aging, positive aging, vital aging, and creative aging); (2) social engagement (eg, active aging, social aging, productive aging, and conscious aging); (3) technology and innovation (eg, smart aging and intelligent aging); and (4) resilience and adaptation (eg, adaptive aging, resilient aging, and robust aging). Although all these concepts are built upon the foundation of health, their differing emphases reflect the multidimensional nature of aging research in academia. For instance, concepts centered on health and well-being focus on enhancing physical health and quality of life, while social engagement concepts underscore the social roles and contributions of older adults.

Nevertheless, despite these expanded areas of focus, practical applications often remain fragmented, limiting their broader impact on societal perceptions of aging. Specifically, the content analysis in this study, which also explored challenges and future trends, revealed significant obstacles to achieving the goals of aging concepts, such as unequal access to health care, technological barriers, and cultural differences. For example, “smart aging” initiatives leveraging digital technology, though beneficial for enhancing independence, face barriers such as limited access and high costs, restricting their reach [65]. Similarly, resilience-oriented concepts that promote coping strategies for psychological and physical challenges often lack widespread implementation and do not significantly influence public policy or community practices [66]. These findings underscore the complexity of effectively implementing aging concepts and point to potential pathways for progress.

To drive a meaningful shift in the negative narrative on aging, interdisciplinary strategies are essential, bridging current gaps to create a cohesive and impactful transformation [67]. Several successful approaches from other domains offer promising models for adaptation in aging research. For example, universal design in urban planning can inform inclusive spaces and housing that support physical and cognitive aging. Lifelong learning platforms from the education sector can promote digital literacy and social engagement. Resilience-building frameworks, developed in clinical psychology and disaster management, offer coping strategies that can be tailored to later-life transitions. Integrating such practices into aging-focused interventions can help bring empowerment-based narratives into real-world application.

These strategies must ensure that technological advancements and global awareness translate into tangible benefits that are accessible, affordable, and inclusive for all [68,69]. Future policies should adopt a nuanced understanding of aging by fostering integrated research that unites medical, social, and technological aspects, thus enabling a holistic perspective that can reshape both academic and societal narratives on aging. For instance, practical strategies like expanding social networks, offering flexible employment, and fostering lifelong learning opportunities are crucial, as they not only enhance older adults’ sense of belonging and mental well-being but also drive a transformative shift in societal perceptions and approaches to aging [70-72]. Without such efforts, there is a risk of reinforcing existing inequalities rather than resolving them. As indicated by the cocitation and collaboration analyses, current aging research remains structurally concentrated within biomedical networks and Western regions, suggesting that future narrative shifts require more interdisciplinary and globally distributed scholarly engagement.

### Limitations and Future Directions

While this study offers valuable insights for both researchers and policy makers, it has certain limitations. The exclusive reliance on the Scopus database may have led to the omission of relevant studies from other sources. Additionally, although multiple analytical tools such as VosViewer and AntConc were used, differences in their functionalities may introduce interpretation biases. Furthermore, this study did not fully explore these aging concepts’ roles in policy-making and cross-cultural contexts, leaving room for future research in these important areas.

Future research could address several areas identified in this study. First, expanding the data sources to include additional databases (eg, Web of Science or PubMed) may broaden disciplinary representation. Second, exploring how aging concepts are implemented and interpreted in policy settings would help assess their practical influence. Third, cross-cultural comparisons could reveal differences in how aging narratives are constructed globally. Finally, mixed method approaches combining bibliometric, linguistic, and qualitative data could provide a more comprehensive understanding of both academic and societal impacts of aging discourse.

### Conclusions

This study comprehensively analyzes 16 aging concepts, elucidating their main focuses, applications, and the challenges and future trends associated with their implementation. The analysis reveals an overall picture of the academic landscape, including the distribution of journals and disciplines, as well as cocitation networks and regional collaborations. “Healthy aging” emerged as the most dominant concept according to its frequency in the 2 datasets. A content analysis further categorized the 16 concepts into four primary groups based on focus and application, enhancing our understanding of their roles: (1) health and well-being (eg, healthy aging, successful aging, optimal aging, positive aging, vital aging, and creative aging); (2) social engagement (eg, active aging, social aging, productive aging, and conscious aging); (3) technology and innovation (eg, smart aging and intelligent aging); and (4) resilience and adaptation (eg, adaptive aging, resilient aging, and robust aging). Notably, “healthy aging” and “creative aging” were the earliest concepts, first appearing in 1962, marking the beginning of an evolving discourse on aging. While these concepts collectively aim to enhance the quality of life for older adults and reshape the negative narrative on aging, progress has been uneven, with many efforts remaining siloed and largely focused on medical aspects. Although there has been a shift toward more holistic perspectives, including social engagement, resilience, and technological adaptation, these approaches have yet to achieve widespread and integrated impact.

This classification was developed through systematic content analysis, grouping concepts by their primary thematic focus and area of application. It serves not only as a descriptive framework but also offers practical relevance. For researchers, it highlights patterns across disciplines and identifies gaps for future study. For policymakers, it enables more targeted approaches—for instance, by aligning interventions with specific conceptual clusters such as promoting social participation or enhancing technological access for older adults. This structured understanding of the aging discourse can guide more coordinated, interdisciplinary strategies in both research and policy development.

This study contributes uniquely to the existing literature in several ways. First, it provides a comprehensive cross-conceptual comparison of 16 aging frameworks, offering a panoramic view that is rarely achieved in prior studies focused on single concepts. Second, by integrating bibliometric mapping with corpus linguistic analysis, it introduces a multimethod approach that captures both the structural distribution and the narrative functions of aging discourse. Third, the study applies a theoretically grounded lens—drawing on narrative theory and the social construction of aging—to assess how conceptual framing influences the persistence or transformation of age-related narratives. Together, these contributions advance a more integrated and reflexive understanding of how academic discourse shapes societal perspectives on aging.

Significant challenges persist, including unequal access to health care, technological barriers, and cultural differences, which complicate the effective implementation of these aging concepts. Addressing these challenges requires coordinated strategies that ensure accessibility, affordability, and inclusivity, translating technological advancements and global awareness into tangible benefits for all older adults. Future policies must be informed by a nuanced understanding of aging, emphasizing preventive health care, personalized interventions, and social and psychological support. Practical measures like expanding social networks, offering flexible employment, and fostering lifelong learning opportunities are essential for promoting a sense of belonging and enhancing mental well-being among older adults.

## Supplementary material

10.2196/72011 Multimedia Appendix 1 Disciplinary distribution of publications.

10.2196/72011 Multimedia Appendix 2 Frequency of 16 aging-related concepts by using AntConc.

10.2196/72011 Multimedia Appendix 3 Comparisons of the frequency of 16 aging concepts in achieving “Age Well.”

## Abbreviations

AI: artificial intelligence
TLS: total link strength
WHO: World Health Organization
