# Supplementary figures and images for: Comparative Analysis of 16 Aging Concepts and Their Influence on Aging Narratives: Bibliometric and Content Analysis
Source: JMIR Aging. 2025 Sep 23;8:e72011. doi: 10.2196/72011 (PMC12605274; doi:10.2196/72011)

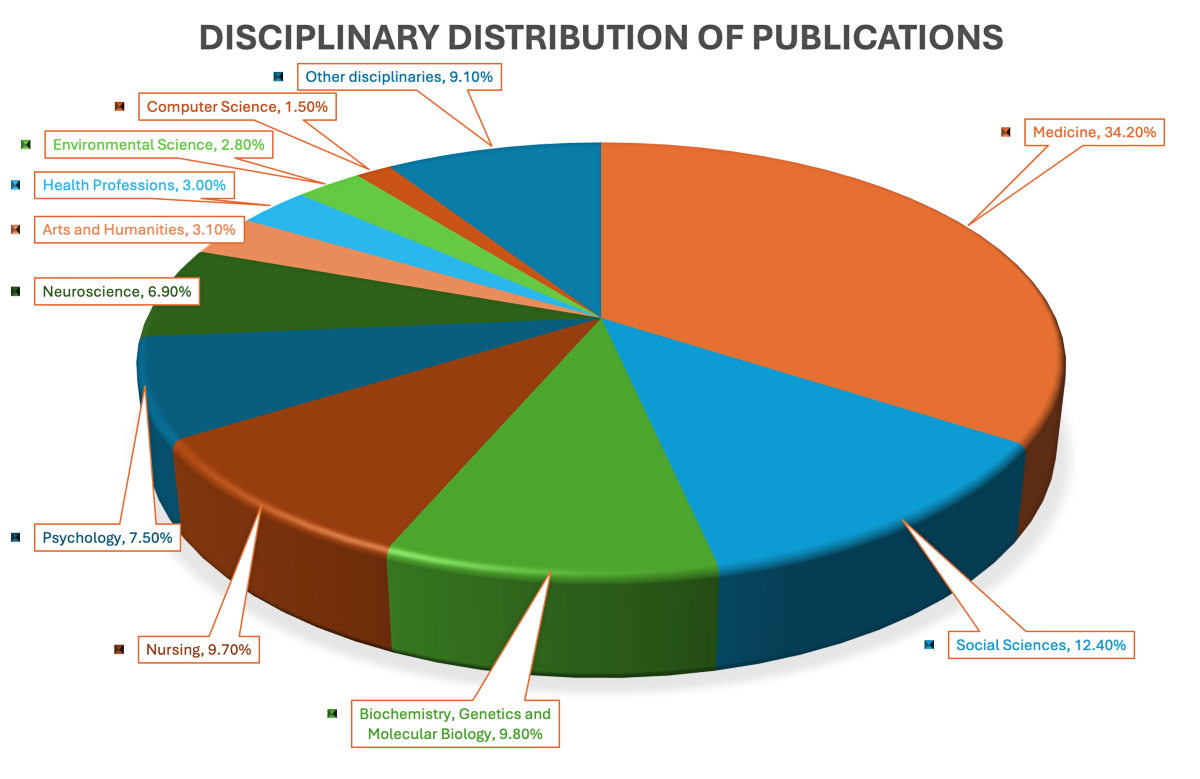

Supplement: Multimedia Appendix 1 [file aging-v8-e72011-s001.png]
