# Supplementary material for: Comparative Analysis of 16 Aging Concepts and Their Influence on Aging Narratives: Bibliometric and Content Analysis
Source: JMIR Aging. 2025 Sep 23;8:e72011. doi: 10.2196/72011 (PMC12605274; doi:10.2196/72011)
Supplement: Multimedia Appendix 3 [file aging-v8-e72011-s003.docx]

| **Ageing concepts** | **Frequency** |
| --- | --- |
| successful aging / successful ageing | 417 |
| healthy aging / healthy ageing | 247 |
| active aging / active ageing | 180 |
| positive aging / positive ageing | 70 |
| productive aging / productive ageing | 14 |
| optimal aging / optimal ageing | 8 |
| vital aging / vital ageing | 6 |
| creative aging / creative ageing | 4 |
| adaptive aging / adaptive ageing | 3 |
| resilient aging / resilient ageing | 1 |
| social aging / social ageing | 1 |
| healthy-active aging / healthy active aging / healthy-active ageing / healthy active ageing | 1 |
| intelligent aging / intelligent ageing | 0 |
| smart aging / smart ageing | 0 |
| conscious aging / conscious ageing | 0 |
| robust aging / robust ageing | 0 |
